# ECG interpretation in athletes by expert sports cardiologists and nonspecialist medical professionals across Europe: a survey from the European association of preventive cardiology

**DOI:** 10.1093/ehjopen/oeag183

**Published:** 2026-10-29

**Authors:** Emmanuel Androulakis, Hamish MacLachlan, Elena Cavarretta, Vassilios S Vassiliou, Dimitra Antonakaki, Rocco Friebel, Meskerem Kebede, Raghav Bhatia, Sabiha Gati, Gherardo Finocchiaro, Michael Papadakis, Silvia Castelletti, Flavio D’Ascenzi

**Affiliations:** Inherited Cardiac Conditions, King’s College Hospital, Denmark Hill, London SE59RS, UK; King’s College London, School of Cardiovascular and Metabolic Medicine and Science, Strand, London WC2R 2LS, UK; Cardiovascular CAG and Cardiology Research Centre, City St. George’s University of London, Cranmer Terrace, London SW17 0RE, UK; Cardiovascular CAG and Cardiology Research Centre, City St. George’s University of London, Cranmer Terrace, London SW17 0RE, UK; Department of Medico-Surgical Sciences and Biotechnologies, Sapienza University of Rome, Corso della Repubblica, 79, 04100 Latina, Italy; Advanced Cardiovascular Therapies Unit, Bambino Gesù Children’s Hospital, IRCCS, Piazza di Sant'Onofrio, 4, 00165 Rome, Italy; Department of Cardiology, Norfolk and Norwich University Hospital, Colney Lane, Norwich, Norfolk NR4 7UY, UK; Norwich Medical School, University of East Anglia, Norwich Research Park, Earlham Road, Norwich NR4 7TJ, UK; Department of Cardiology, Onassis Cardiac Surgery Centre, Athens, Greece; Department of Health Policy (Cowdray House 2.09), The London School of Economics and Political Science, Houghton Street, London WC2A 2AE, UK; Department of Health Policy (Cowdray House 2.09), The London School of Economics and Political Science, Houghton Street, London WC2A 2AE, UK; Cardiovascular CAG and Cardiology Research Centre, City St. George’s University of London, Cranmer Terrace, London SW17 0RE, UK; Hull Royal Infirmary, Hull University Teaching Hospitals NHS Trust, Anlaby Road, Kingston-upon-Hull HU3 2JZ, UK; Royal Brompton Hospital, Guy’s and St Thomas’ National Health Service Foundation Trust and National Heart and Lung Institute, Imperial College, Sydney Street, Chelsea, London SW3 6NP, UK; St. George’s University Hospitals NHS Foundation Trust, Blackshaw Road, Tooting, London SW17 0QT, UK; Cardiovascular CAG and Cardiology Research Centre, City St. George’s University of London, Cranmer Terrace, London SW17 0RE, UK; St. George’s University Hospitals NHS Foundation Trust, Blackshaw Road, Tooting, London SW17 0QT, UK; Cleveland Clinic London, 33 Grosvenor Place, London SW1X 7HY, UK; Department of Sports Cardiology, University Hospital Santa Maria Della Misericordia, Piazzale Santa Maria Della Misericordia 15, Udine 33100, Italy; Department of Medical Biotechnologies, Sports Cardiology and Rehab Unit, University of Siena, Policlinico Santa Maria alle Scotte Viale Mario Bracci, 16, 53100 Siena, Italy

**Keywords:** athletes, ECG interpretation, T-wave inversions, Preparticipation screening, Cardiomyopathy, Arrhythmia syndrome

## Abstract

**Aims:**

We aimed to evaluate the real-world use of contemporary international ECG interpretation criteria in athletes, and to compare practices, clinical decision-making, and perceived barriers between specialist and nonspecialist professionals across Europe.

**Methods and results:**

Two sets of structured questionnaires were developed and distributed to either sports cardiologists or nonspecialist professionals. Knowledge of international guidelines, experience in ECG interpretation, and confidence levels in clinical decision-making across various clinical scenarios were evaluated. An open-ended questionnaire was also developed to explore aspects of ECG interpretation, medicolegal issues, relevant legal frameworks across European countries, and personal thoughts on possible future revisions to the documents. The questionnaires were revised and edited by expert sports cardiologists, affiliated with EAPC. Data were analysed both quantitatively and qualitatively. A total of 242 participants across Europe were included; 66% were male and 57% worked in public hospitals. Nonspecialists demonstrated uncertainty in standard definitions, classification systems, and key applications of ECG criteria. These findings highlight variability in preparticipation screening (PPS) practices and areas of uncertainty in clinical decision-making, alongside concerns regarding access to specialist services. Notably, 25% of specialists favoured a flexible interpretative approach beyond current standard criteria. The majority supported the need for updated guidance and improved description of specific ECG indices, including ventricular ectopy (83%), QRS fragmentation (83%), and low-amplitude QRS complexes (78%), alongside recognition of the potential role of artificial intelligence–based models. Gaps in knowledge, training, and practice were identified, and both groups emphasized medicolegal and pathway challenges.

**Conclusion:**

This international survey provides a contemporary real-world overview of ECG interpretation and PPS practice in athletes across Europe and revealed substantial heterogeneity in access to services, training, interpretation uncertainty, referral pathways, and medico-legal frameworks.

## Graphical Abstract

**Graphical Abstract:**
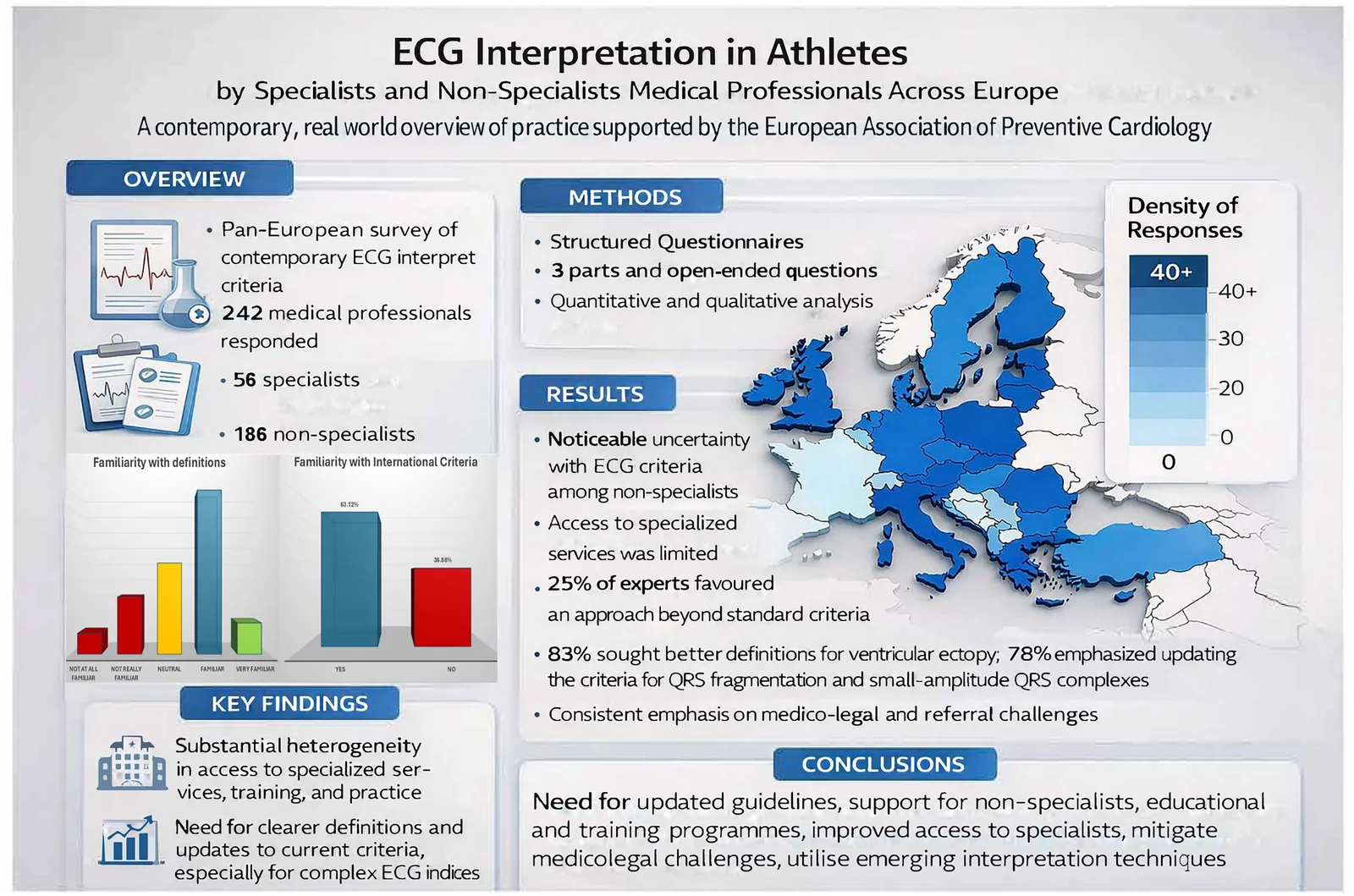

## Introduction

The resting 12-lead electrocardiogram (ECG) enhances the detection of cardiovascular conditions associated with sudden cardiac death in paediatric and adult athletes.^1,2^ Regular intensive exercise induces a range of physiological adaptations, collectively described as the athlete's heart, which may manifest as ECG changes that overlap with features of underlying cardiac disease.^3^ This creates a diagnostic challenge, particularly in asymptomatic individuals.

To address this, international ECG criteria have been developed to distinguish physiological adaptations from pathological findings and to standardize clinical evaluation. Over the past 15 years, these criteria have undergone successive refinements based on large-scale screening studies and expert consensus, improving diagnostic accuracy and reducing unnecessary downstream investigations.^4–9^ Despite these advances, uncertainty persists in the interpretation of specific ECG patterns, selected athlete populations, and subsequent management decisions.^10,11^

With updated ECG criteria anticipated, understanding current real-world practice, knowledge gaps, and barriers to implementation is essential. This study therefore aimed to evaluate ECG interpretation practices among specialist and nonspecialist clinicians across Europe and to identify areas of variability that may influence future guideline uptake.

## Methods

The survey was conducted from March to June 2025. Medical professionals from European Association of Preventive Cardiology (EAPC)-registered units were invited to participate through multiple channels, including EAPC newsletters, social media platforms, direct email invitations, and the EAPC Ambassadors network. This approach enabled broad participation across Europe and inclusion of clinicians involved in athlete care across a range of clinical settings. As recruitment was conducted through EAPC-affiliated channels, the sample may be enriched for clinicians with a preexisting interest in sports cardiology.

The survey was structured in three parts. The first part included demographic information such as age, country of practice, practice setting (public or private), and clinical specialty. Participants were asked to self-identify their primary clinical interest and practice. Specialists were defined as clinicians whose primary clinical focus is sports cardiology. Nonspecialists were defined as medical professionals involved in the care of athletes whose primary clinical practice lies outside sports cardiology, including general cardiologists, physicians, and general practitioners.

Group classification was based on self-identification of primary clinical interest and practice rather than formal accreditation or predefined objective criteria. Specialists were defined as clinicians whose primary clinical focus is sports cardiology. Nonspecialists were defined as medical professionals involved in the care of athletes whose primary clinical practice lies outside sports cardiology, including general cardiologists, physicians, and general practitioners.

Subsequently, two sets of structured questionnaires were developed and distributed to either Sports Cardiology specialists or nonspecialist senior medical professionals who manage athletes in their routine clinical practice (see Supplementary material online, *Appendix A*). These encompass multiple sections on information about the country/area, Sports Cardiology centres/practitioners, level of confidence in ECG interpretation, knowledge and understanding of international guidelines, experience in ECG interpretation, and confidence levels in clinical decision-making across various clinical scenarios involving ECG variations. An open-ended questionnaire was also developed to explore specific aspects of ECG interpretation, such as ventricular ectopy, as well as medicolegal elements, the relevant legal framework across European countries, and personal thoughts on possible future revised documents. The questionnaires were revised and refined through iterative review by expert sports cardiologists affiliated with the EAPC to ensure clinical relevance, clarity, and content validity. Although formal validation procedures (such as pilot testing or assessment of reliability and internal consistency) were not performed, the survey was designed to reflect key domains of clinical practice and decision-making.

### Qualitative and quantitative analysis

Free-text and quantitative responses were analysed to explore clinician perceptions (Both Specialists and nonspecialists) across multiple domains. Two researchers from the London School of Economics (MK and RF) conducted a qualitative analysis of the open-ended survey responses using an inductive-deductive thematic approach. The researchers were blinded to respondents’ identities beyond their clinician role (specialist or nonspecialist). Responses were imported into Microsoft Excel (Microsoft 365 Enterprise) for data organization and systematic coding. Both researchers independently reviewed all free-text responses and conducted an initial pilot coding on a subset of entries to establish consistency in interpretive approach. MK and RF independently coded all remaining responses on NVIVO QSR International using the agreed codebook, with discrepancies resolved through discussion and consensus. Key concepts within each code were then synthesized to identify overarching themes, supported by illustrative quotes drawn directly from participants’ survey responses.

Categorical variables were expressed as counts and percentages, while continuous variables with a normal distribution were reported as means ± standard deviation (SD). A *P*-value of <0.05 was considered statistically significant, and all analyses were performed using SPSS version 21.0 (SPSS Inc., Chicago, IL, USA).

### Purpose

The purpose of the survey, which included 40 multiple-choice and open-ended questions in total, was to explore (i) in-depth understanding of current guidance of ECG interpretation in athletes by nonexpert medical professionals, (ii) variations in practice between Sports Cardiologists and nonspecialist medical professionals, (iii) variations in practice across European countries or specific regions with variable accessibility to Sports Cardiology services, (iv) potential impact on athletic individuals management, (v) reveal ECG findings that are still equivocal and create variability in practice even within Sports Cardiology specialists, (vi) discuss the potential necessity for revision of those criteria on a dedicated document across International professionals, incorporating new criteria or even novel, more advanced techniques of ECG interpretation such as artificial intelligence (AI), currently available. Free-text and quantitative responses were analysed to explore clinician perceptions (Both Specialists and nonspecialists) across multiple domains.

## Results

### Participant background

A total of 242 participants across Europe responded to the Survey. The vast majority of participants were aged 30–60 (85%). Male physicians accounted for 66% of respondents, with female physicians comprising 34%, indicating relative under-representation, which might reflect overall gender differences in the field of sports cardiology and medicine. Participants were drawn from a wide range of European countries, with heterogeneous geographic representation across regions (*Figure 1*). Overall, most European Society of Cardiology (ESC) members and ESC-affiliated countries were represented. The distribution of respondents across countries was heterogeneous, and the number of participants per country varied, limiting the ability to draw country-specific conclusions. Fifty-seven per-cent of participants work in a public hospital, 20% in a private institution, and 17% have their own private practice.

**Figure 1:**
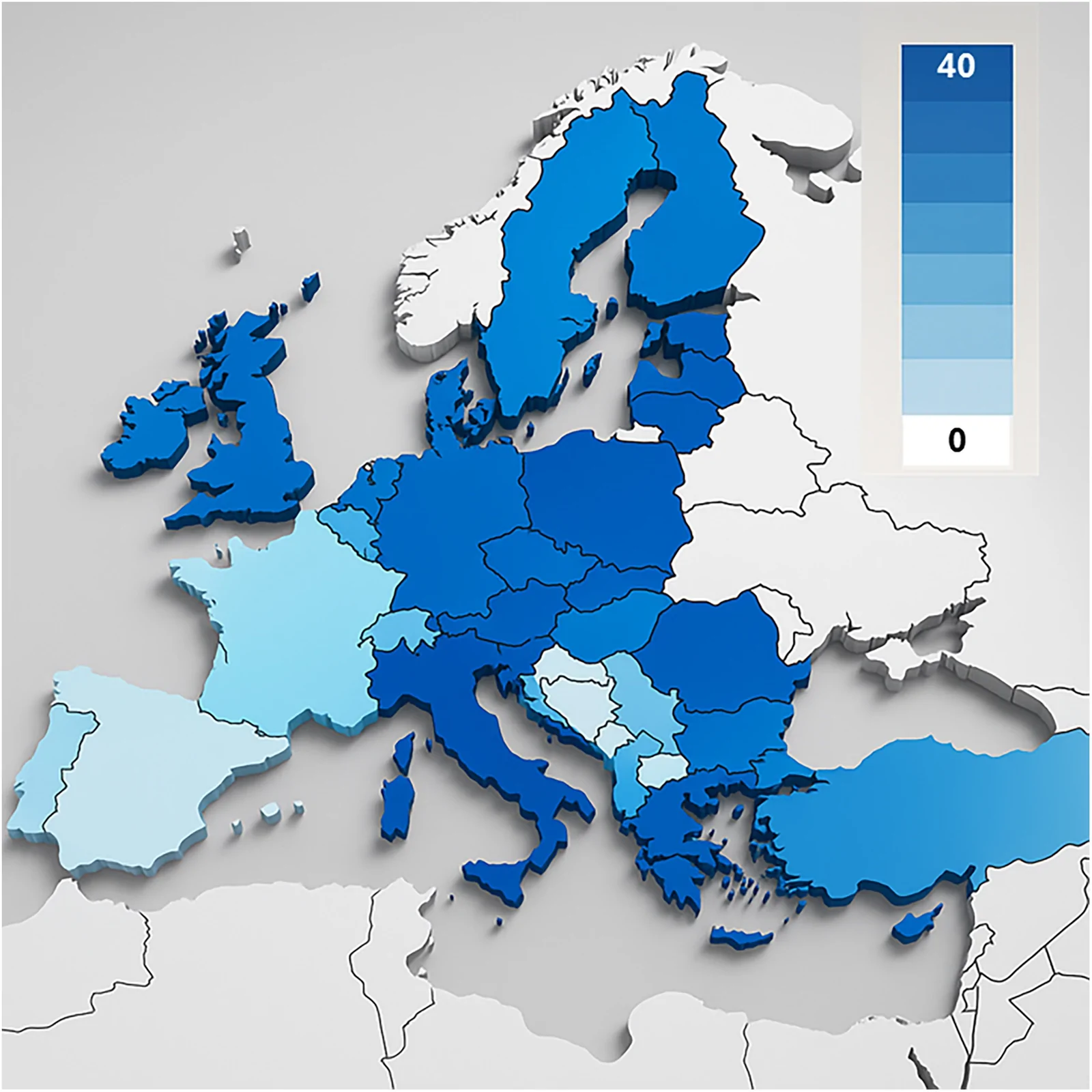
Participants’ background representing the European association of preventive cardiology (EAPC)-affiliated countries.

### Access to services

The majority of nonspecialist respondents (52%, 81/156) reported active involvement in the management of athletes. Referral practices were heterogeneous: 11% referred all athletes, 10% most athletes, 5% approximately half, 54% few (77/141), and 21% none (29/141).

When considering service infrastructure, the majority of nonspecialist participants were general cardiology physicians, other cardiology specialities such as arrhythmia and heart failure, alongside other physicians, and general practitioners. A prevalent answer (57%, 89/156) was that there is no sports cardiology centre in their region, while 43% (66/155) responded that there is no sports specialist in their area. Despite this, the majority (52%, 81/156) of nonspecialists manage professional athletes in a variety of sports. Interestingly, the majority of respondents refer either no athletes or only a few to further assessment, while only 10% refer all athletes to specialists. Access to sports cardiology centres differed significantly between groups, with 77% (37/48) of specialists reporting availability compared with substantially lower access among nonspecialists (*P* < 0.05) (*Figure 2*). Notably, only a proportion of sports specialists across Europe work in dedicated sports cardiology centres.

**Figure 2:**
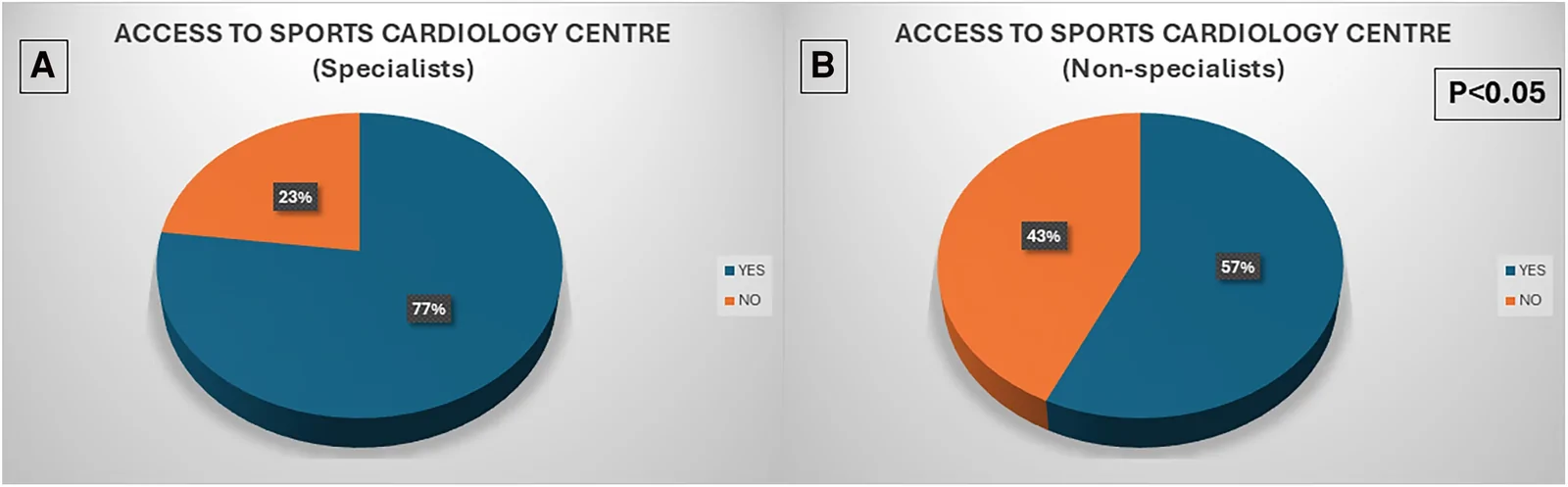
Access to sports cardiology centres as reported by sports specialists (*A*), and nonspecialists (*B*). This was significantly different (*P*  *<*  *0.05*), indicative of possible limitations in access to specialist services.

### Familiarity with definitions and contemporary ECG criteria among nonspecialists

Familiarity with sports classification systems varied across professional societies: 43% reported familiarity with the (European Association of Preventive Cardiology) EAPC/ESC classifications, 2% with the Association for European Paediatric and Congenital Cardiology (AEPC), and 19% with the American Heart Association/American College of Cardiology (AHA/ACC).

Fewer than half of respondents reported familiarity with the definition of an athletic individual and its subtypes (*Figure 3A*). Awareness of the 2017 International ECG Criteria was reported by 63% of respondents (*Figure 3B*), of whom the majority indicated routine use in clinical practice. However, confidence in interpreting an athlete's ECG was reported only by 51%, with 32% reporting neutral confidence and 8% low or no confidence.

**Figure 3:**
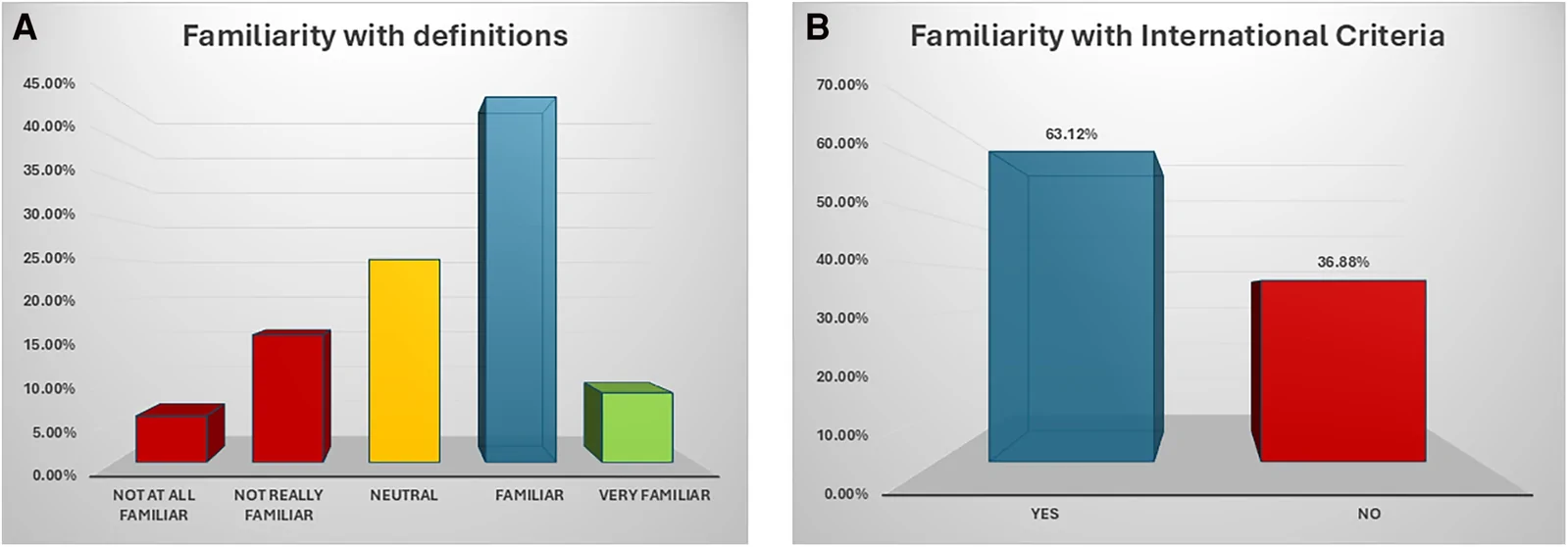
Familiarity with definitions of athletic individuals, and different types of exercise (*A*). Familiarity with International ECG criteria (*B*) in nonspecialists.

Nonspecialists demonstrated limited familiarity with international consensus standards, particularly ‘borderline’ ECG findings, with 52% reporting low or no familiarity (*Figure 4*). Similarly, understanding of key concepts such as athletic adaptation, its overlap with cardiac pathology, and associated ECG changes was also limited (*Figure 5*). Nonspecialists also demonstrated limited familiarity with specific repolarisation variants in anterior leads that may warrant specialist referral. Substantial variability was observed in the recognition of anterior T-wave inversion patterns, including juvenile patterns (58%), patterns in African Caribbean athletes (37%), Caucasian females (40%), and patterns suggestive of arrhythmogenic right ventricular cardiomyopathy (55%) (*Figure 6*).

**Figure 4:**
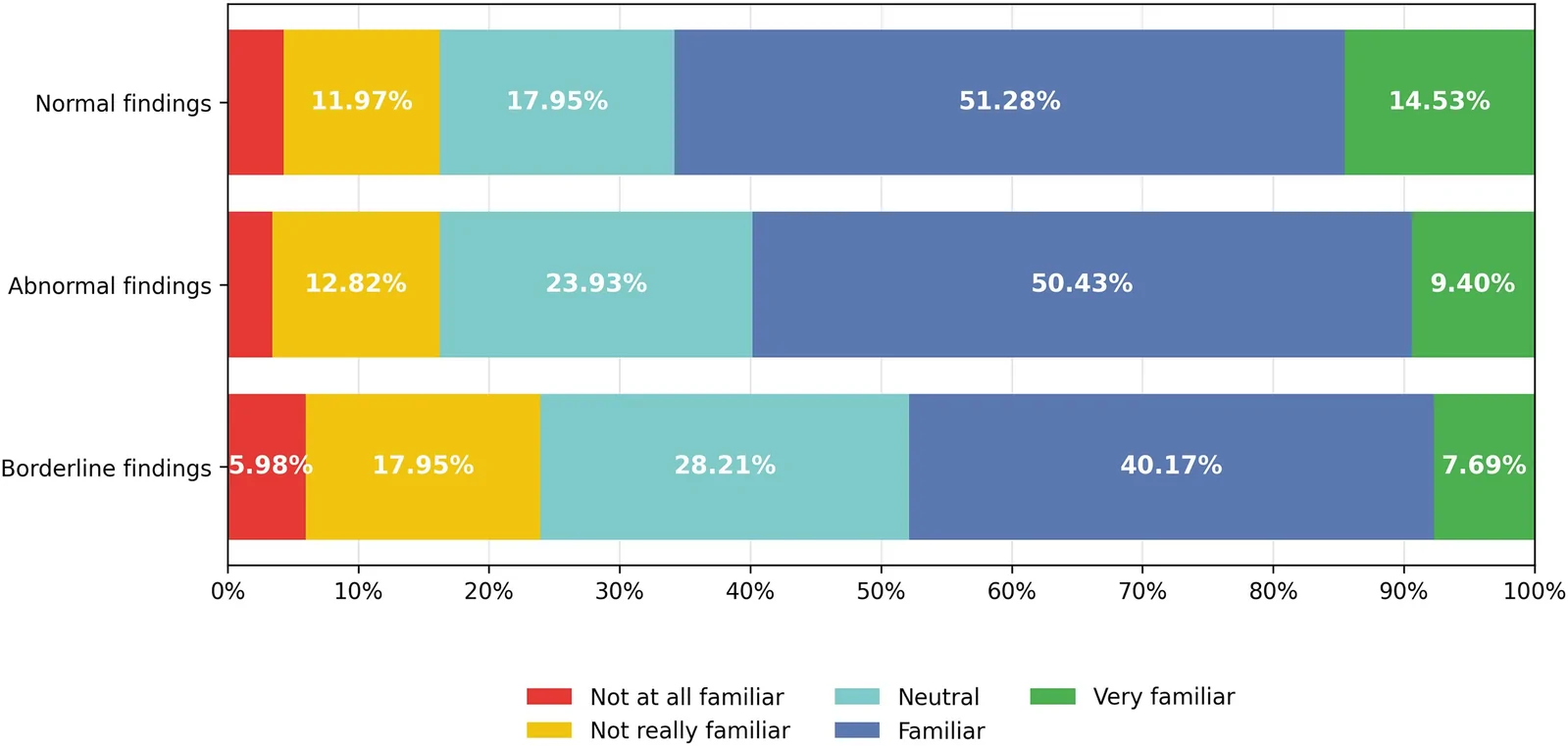
Familiarity with the international consensus standards; ‘normal’, ‘borderline’ and ‘abnormal’ findings in nonspecialists.

**Figure 5:**
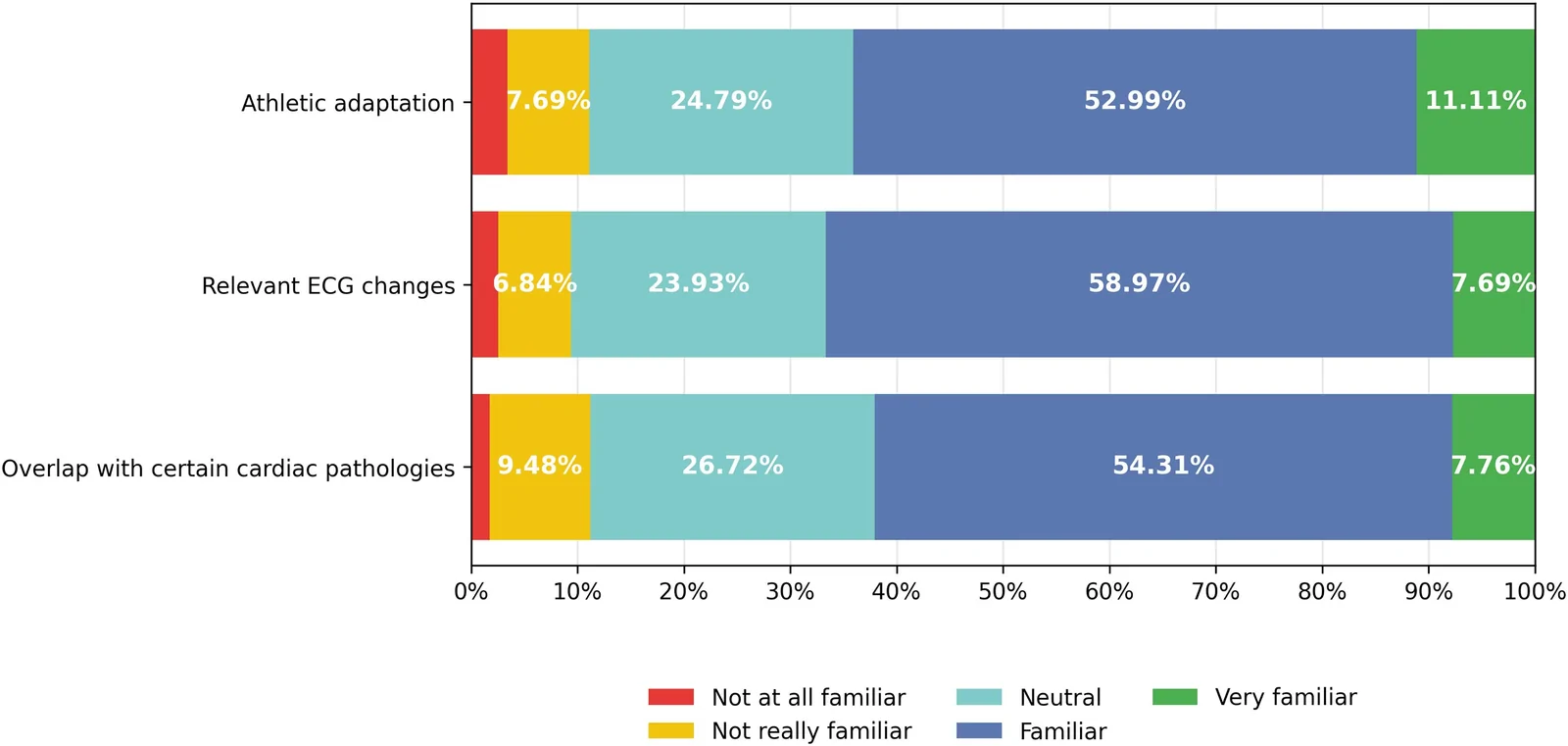
Familiarity with concepts of ‘athletic adaptation’, ‘overlap of athletic remodelling’ with cardiac pathology, and ‘athletic remodelling’-related alterations on ECG by nonspecialists.

**Figure 6:**
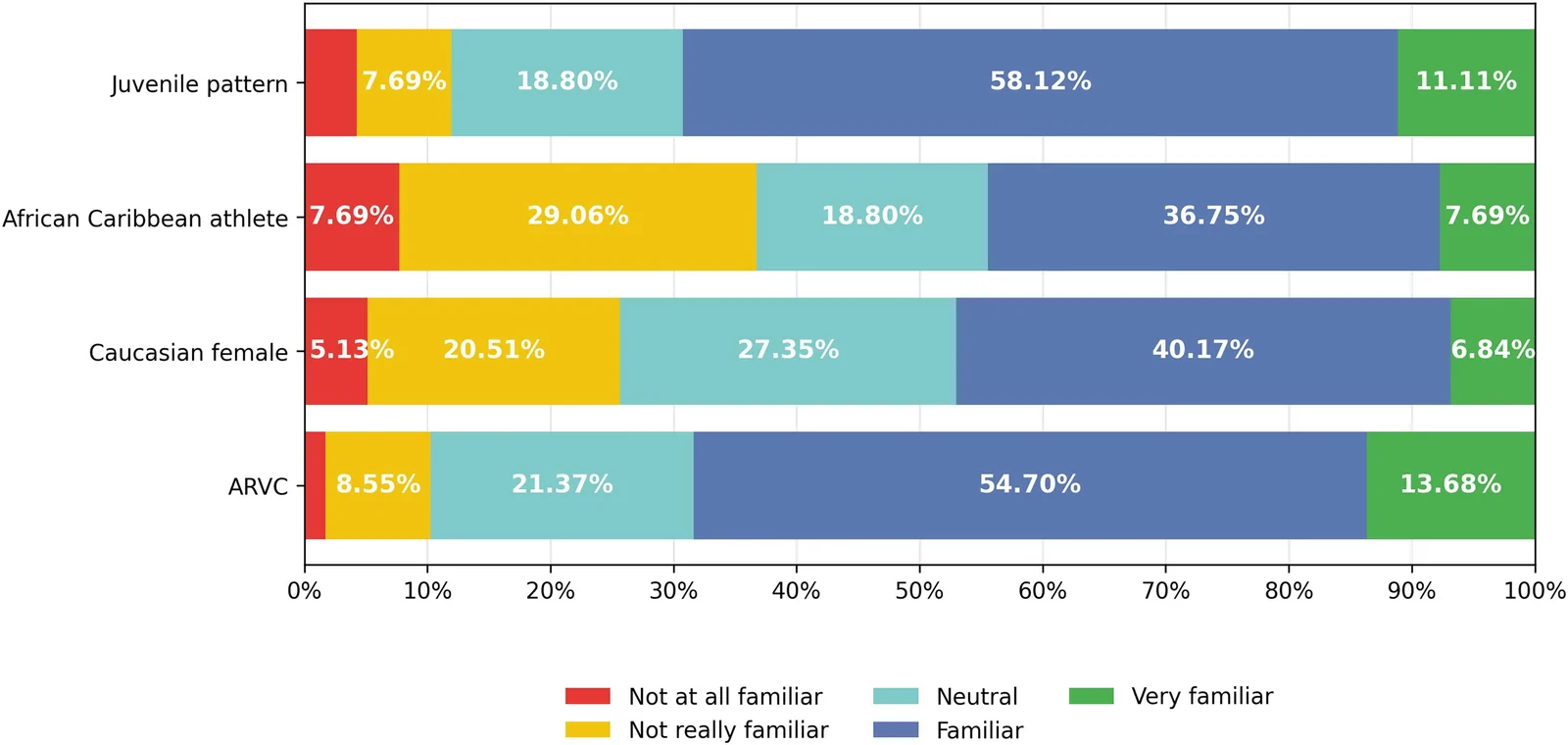
Familiarity with concepts of different variations of anterior repolarisation changes reported by nonspecialists.

The overwhelming majority (95%) of participants would use a simple international reference medical guide for the ‘nonspecialist’ to facilitate management and potential referral to sports specialists, if this had been available. The legal framework does not seem to be efficient for nonspecialist clinicians to make decisions about excluding athletes from participation in their country (72% were supportive of this opinion or unsure). The specialists answered the same question, and a comparable percentage (73%, NS) also agreed with this view.

### Current and future applications of ECG criteria

Among specialists in routine practice, 75% (39/52) reported using the 2017 International ECG Criteria, whereas 25% favoured a more flexible interpretative approach. Specialists demonstrated marked variability in their assessment of the adequacy of the International ECG Criteria across multiple ECG findings. Only 70% considered anterior T-wave inversion patterns and their management to be adequately described, with similar proportions reporting adequate guidance for bundle branch block morphologies (70%), notably ventricular ectopic beat morphology and management (38%).

Several ECG indices were predominantly perceived as insufficiently addressed in the current criteria, including QRS fragmentation (83%, 43/52), low-voltage QRS complexes (78%), and nonspecific intraventricular conduction delay (64%), with respondents reporting either inadequate description or uncertainty. Uncertainty also extended beyond specific ECG patterns to population subgroups: only 39% of specialists reported having adequate information to guide ECG interpretation in master athletes, while 42% considered the guidance insufficient, and 19% were unsure.

When managing an athlete with an abnormal ECG, 48% reported adhering to the recommended further evaluation outlined in the International Criteria, while 52% preferred an experience-based approach to requesting additional investigations. When asked about the need to revise the 2017 International ECG Criteria, the vast majority 83% of respondents supported revision, 13% did not, and 4% were unsure.

With respect to emerging technologies, 74% of respondents felt that artificial intelligence could be useful in ECG interpretation in clinical practice, while 13% disagreed and 13% were unsure; similarly, 65% supported inclusion of recent data on artificial intelligence–based ECG interpretation in future guideline documents.

As already mentioned, the majority (75%) specialists use the standard division of findings into ‘normal’, ‘abnormal’, and ‘borderline’ according to the International criteria^5^; however, one-fourth of them adopt a more flexible approach to ECG interpretation. When managing athletes with abnormal ECG findings, a substantial proportion of specialists reported adopting a flexible approach to further investigation. This likely reflects the application of expert clinical judgement in complex or borderline scenarios, particularly where guideline recommendations are less prescriptive or where individual patient factors require tailored decision-making. Such variability may therefore represent appropriate individualization of care rather than deviation from best practice.

Quite relevant to this, the specialists think that certain ECG indices are not adequately described in the International criteria. More specifically, the vast majority (74%) think QRS fragmentation requires further clarification; similarly, low voltage QRS and nonspecific intraventricular conduction delay are not adequately described. Of note, specialists also believe there is insufficient evidence to guide ECG interpretation in master athletes.

### Educational and training needs in view of the new era

Most specialists agreed or strongly agreed (60%, 29/48) that a significant degree of interobserver variability exists among sports specialists in ECG interpretation. Despite this, 58% reported that athletes’ ECG interpretation and management are primarily led by sports specialists in their country, and among these respondents, 32% considered that sports specialists possess inadequate training and experience to fulfil this role. In this context, emerging technologies were viewed as a potential means of improving consistency in ECG interpretation: 74% of respondents felt that artificial intelligence could be useful in ECG interpretation in clinical practice.

Almost 60% of the specialists agree that there is significant interobserver variability among them, and they acknowledge that a substantial proportion of ECG interpretation and management in their country (35%) is handled by nonspecialists. An interesting, relevant question about whether sports specialists in their country have adequate training and experience elicited a negative response from 32% of respondents. It is also worth emphasizing that the vast majority of specialists (83%, 43/52) believe that the 2017 International Criteria should be revised. Relevant to this, the majority (66%) also thinks a possible new guideline document would preferably include emerging data from the use of artificial intelligence (AI) in ECG Interpretation, based on the thought that AI could indeed be used in ECG interpretation in clinical practice by the majority (74%) of the participants.

### Medico-legal uncertainty and lack of regulatory frameworks

Medico-legal uncertainty was a dominant finding across both groups. Among nonspecialists, over half (54%) reported a lack of clear national regulation, while similar concerns were expressed by specialists (62%), with 42% highlighting medico-legal risks related to participation decisions. These findings reflect widespread ambiguity regarding clinical responsibility and legal accountability in athlete screening.

Free-text responses reinforced this uncertainty, with participants emphasizing inconsistent governance across healthcare systems and sporting organizations. One respondent noted that ‘there is no legal framework; each sport may have its own regulations, and some do not require cardiac screening/sign-off,’ illustrating the fragmented and nonstandardized nature of current practice.

### Variability in service organization and referral pathways

Substantial variability in service organization and referral pathways was reported. Nonspecialists frequently described limited access to sports cardiology expertise, with 55% reporting restricted access to specialist services and 34% expressing uncertainty regarding responsibility when advising on participation. These findings highlight structural limitations in service provision across regions.

Qualitative responses further illustrated this variability, particularly in relation to decentralized models of care and inconsistent implementation of screening pathways. For example, one participant stated that ‘PPS is performed at the primary health care setting by primary care physicians who are family medicine specialists or general practitioners, and although there are national guidelines, there is no legal regulation for this process.’ Specialists similarly described variability across sporting organizations, reflecting fragmentation in referral pathways and lack of standardization.

### Practical challenges in clinical decision-making

Practical challenges in managing athletes with potential cardiac abnormalities were frequently reported, with differences observed between groups. Nonspecialists highlighted limited access to expertise and external pressures, while specialists identified gaps in structured clinical pathways. Among specialists, 52% reported lack of evidence-based exercise prescription pathways, and 44% described diagnostic uncertainty in cases with abnormal ECG findings but normal imaging.

These challenges were reflected in qualitative responses, with nonspecialists emphasizing system-level limitations and uncertainty, as summarized by the statement ‘lack of experts and adequate sports cardiology centres, law not clear, liability not clear.’ Specialists, in contrast, focused on clinical ambiguity, noting that ‘exercise prescription in athletes with pathological findings but no clear diagnosis’ remains a key difficulty in practice.

### Diagnostic uncertainty and interpretative ambiguity

Diagnostic uncertainty emerged as a key theme, particularly in relation to ECG interpretation and clinical decision-making. Among specialists, no universally accepted threshold for ventricular ectopy was identified, with 56% favoring a context-dependent approach and 44% rejecting fixed thresholds. In addition, the majority (83%) supported revision of the current ECG criteria, reflecting perceived gaps in existing guidance.

Qualitative responses supported these findings, highlighting reliance on clinical judgement and variability in interpretation. This was illustrated by the view that there is ‘no fixed threshold — depends on symptoms, burden, and athlete context.’ Participants also emphasized uncertainty in relation to emerging or less well-defined ECG indices, reinforcing the need for clearer, evidence-based guidance.

## Discussion

This international Survey provides a contemporary real-world overview of how ECG interpretation and PPS in athletes is currently practised across Europe and reveals substantial heterogeneity in knowledge, referral pathways, training structures, and medico-legal frameworks. Important findings emerged across both specialist and nonspecialist clinicians, highlighting variability in the consistency of cardiovascular screening in athletic populations. As a descriptive survey study, these results reflect clinician-reported practices and perceptions rather than clinical outcomes. This Survey is particularly timely, as it captures uncertainties immediately before the publication of the updated International ECG Criteria. By mapping current gaps in knowledge, training, and management practice, these findings provide an essential clinician-informed justification for evolving the new criteria towards a more comprehensive framework that integrates ECG interpretation with pragmatic clinical management recommendations. Further tangible suggestions and proposals to potentially overcome barriers and obstacles relevant to these findings are highlighted throughout and could be utilized to facilitate further discussion.

A key finding of this study is that the majority of nonspecialists do not regularly refer athletes for specialist sports cardiology assessment. This appears to be driven, at least in part, by structural limitations in service provision, as nearly half of respondents reported no access to a sports cardiologist in their region and up to 60% reported no recognized sports cardiology centre. These survey findings demonstrate a mismatch between clinical exposure and reported specialist availability. In our interpretation, this may represent an important gap in healthcare delivery and supports consideration of structured referral networks. These findings reinforce the urgent need for structured referral networks, investment in regional sports cardiology services, and scalable solutions to bridge expertise gaps, particularly in underserved regions.

Familiarity with sport classification systems was also limited among nonspecialists, with nearly half reporting no knowledge of the ESC classification of sports. This deficit has direct consequences for clinical risk stratification and exercise prescription, which are fundamentally classification-dependent.^12^ For example, individuals with arrhythmogenic cardiomyopathy may be eligible for low-dynamic, skill-based sports but face significantly increased arrhythmic risk with sustained endurance activity. Although the clinical consequences of this knowledge gap were not assessed in the present survey, we speculate that limited familiarity with sport classification could affect the precision of eligibility decisions and exercise advice.

Of particular concern, more than one-third of nonspecialists reported unfamiliarity with contemporary ECG standards, despite many managing athlete ECGs independently and referring to specialist services. This creates a dual clinical risk, with false positive ECG interpretations leading to unnecessary anxiety, investigation, and healthcare expenditure. In contrast, false negative interpretations delay diagnosis and miss opportunities for timely risk-modifying interventions to prevent sudden cardiac death. Interobserver variability in ECG interpretation in athletes is well recognized, particularly between experienced and less experienced clinicians.^10,13^ Our findings further highlight the vulnerability of ECG-based screening when applied without sufficient training. Collectively, these data strongly support the expansion of structured educational initiatives, including validated online ECG training platforms,^14^ standardized teaching within cardiology and sports medicine curricula, and case-based continuing professional development.

An important observation is the discrepancy between reported familiarity with contemporary ECG criteria and relatively modest confidence in ECG interpretation among nonspecialists. The survey identified a discrepancy between reported familiarity with contemporary ECG criteria and confidence in their application. While the underlying reasons were not directly assessed, one possible explanation i**s** the difference between theoretical awareness of guideline frameworks and their practical application in complex or borderline scenarios. Our findings support this interpretation, as nonspecialists reported particular uncertainty in specific domains such as borderline ECG findings, repolarisation variants, and differentiation between physiological adaptation and pathology. These results suggest that familiarity with guidelines alone may be insufficient to ensure confidence in clinical decision-making, highlighting the need for more targeted, case-based training and practical implementation tools. EAPC provides an e-learning platform with sports cardiology clinical cases and relevant resources on the website. Further proposals are currently under consideration and open for discussion, such as the creation of an online platform hosting a ‘hub’ of expert cardiologists, broadly accessible to nonspecialists, to provide expert opinions on challenging cases.

Beyond nonspecialist education, this study also highlights fundamental challenges in defining and training sports cardiology specialists. At present, there is no uniform European definition of sports cardiology expertise based on formal training duration, certification, or legal recognition. This heterogeneity complicates referral pathways, service organization, and even the classification of specialists within research. Recent EAPC initiatives, including certification in preventive cardiology and the evolving role of accredited regional centres, represent necessary steps towards standardizing expertise and ensuring equitable access to specialist care. However, there are few formal higher education programmes in sports cardiology in Europe (such as in the UK and Italy), which are clearly insufficient to meet the needs of a growing population of athletes with cardiovascular disease and the expanding workforce required to manage them effectively. In addition, guided by the American College of Cardiology (ACC) and the ESC frameworks, sports cardiology centres have designed a curriculum integrated into the third year of the general cardiology fellowship. A rigorous, nonstandard fellowship in sports cardiology appears feasible within existing fellowship structures.^15^ We believe these initiatives are extremely important as they can improve patient safety. Existing programmes can be further supported by the ESC, and national societies could work to establish new initiatives across Europe to build a robust framework that ensures high-standard training and optimal clinical management in paediatric and adult populations.

A useful parallel exists in the development of sport and exercise medicine as a recognized medical specialty, as it is already the case in certain countries. Following years of informal practice by interested physicians from diverse backgrounds, sport and exercise medicine achieved full specialty recognition in 2005, supported by the establishment of a national faculty, a defined curriculum, and a structured postgraduate training pathway.^16^ This model enabled doctors to enter dedicated training programmes and acquire specialist competence through a regulated and standardized process. A similar approach could now be considered for sports cardiology across Europe, whereby cardiology trainees may subspecialise in sports cardiology during their specialist training through defined rotations, core competencies, and formal assessment.^17^ Such a pathway would promote a sustainable solution to current workforce limitations, improve uniformity of expertise, and ensure that the growing population of athletes with cardiovascular disease has access to appropriately trained specialists. This is particularly relevant given this Survey's results, which highlight variability in interpretation even among expert sports cardiologists. These findings our supported by a recent study has also demonstrated considerable variability among sport physicians in defining abnormal ECGs, consistent with the findings of the present study.^13^

Furthermore, specialists highlighted several ECG patterns that remain insufficiently characterized, including low QRS voltage, QRS fragmentation, nonspecific intraventricular conduction delay, and the interpretation of ventricular ectopy based on morphology and burden. The survey demonstrated substantial variability in the interpretation of these findings. We interpret this heterogeneity as potentially reflecting both limitations in the available evidence and the limited representation of these indices in earlier guideline documents. Although evolving data have improved understanding of the clinical significance of these ECG features, particularly in relation to cardiomyopathy and arrhythmic risk, their interpretation in athletes remains complex and incompletely defined. In this context, these ECG indices are increasingly recognized in light of emerging contemporary evidence, although this has not yet been fully incorporated into guideline frameworks. This is reflected in the heterogeneity of responses observed among specialists in this study, particularly in relation to ventricular ectopy, where no consistent thresholds or unified approach were identified.

In parallel, specialists expressed uncertainty regarding ECG interpretation in master athletes, paediatric populations, women, and ethnically diverse athletes. These ECG patterns and populations remain underrepresented in existing datasets but are rapidly expanding in clinical practice. Based on the above, there is a strong consensus among sports cardiologists that the 2017 International ECG Criteria should be revised. Although the reasons underlying this view were not formally explored, this may reflect the accumulation of new evidence since their publication and the need for more granular, population-specific guidance.^18–26^

Moreover, the Survey also exposes profound heterogeneity in medico-legal frameworks governing athlete screening and clearance. Both specialists and nonspecialists reported uncertainty regarding legal responsibility, acceptable medical risk, and liability in eligibility decisions. In many regions, cardiac screening is decentralized to primary care or sports organizations without uniform regulatory oversight. The survey demonstrated substantial heterogeneity and uncertainty regarding medico-legal frameworks. Such variation could potentially contribute to professional uncertainty, defensive practice, or inconsistent clearance and restriction decisions. Possibly, a pan-European initiative led by the responsible international committees and the European sports medicine associations, supported by the national societies and governments, could mitigate these issues and establish more unified standards of practice across Europe.

The anticipated role of artificial intelligence (AI) in ECG interpretation emerged strongly from both specialist and nonspecialist respondents. While enthusiasm for AI-supported ECG analysis was high, most clinicians framed AI as a potential decision-support tool rather than a replacement for clinical expertise. These findings reflect clinician perceptions rather than evidence of clinical effectiveness. At present, although numerous condition-specific AI models exist,^27–30^ robust validation in diverse athlete populations remains limited. In addition, important considerations regarding regulatory approval, integration into clinical pathways, and real-world implementation remain to be addressed.

## Limitations

This study has several limitations. As with all survey-based research, participation varied substantially between countries, and in some regions, the number of respondents was small. As a result, the findings may not accurately reflect national practices in individual countries but instead provide an overview of broader regional trends in ECG interpretation and athlete management. Selection bias is also likely, as recruitment was conducted through EAPC-affiliated channels, potentially favouring clinicians with an existing interest in sports cardiology. This may limit the generalisability of the findings to the broader population of clinicians involved in athlete care. In addition, a formal response rate could not be determined due to the distribution strategy, which relied on open invitations through multiple platforms. Although a substantial proportion of respondents were affiliated with European Society of Cardiology bodies or had a declared interest in sports cardiology, this did not uniformly translate into greater familiarity with international ECG criteria, suggesting that knowledge gaps persist even among motivated clinicians.

The Survey relied on self-reported practices and confidence, which may not fully reflect real-world clinical behaviour and are subject to recall and social desirability bias. Moreover, **s**elf-reported expertise and practice patterns may not necessarily reflect actual clinical competence or behaviour.

The questionnaire was not formally validated, and no formal assessment of reliability or internal consistency was undertaken. As such, responses to subjective constructs such as confidence and familiarity should be interpreted with caution. Furthermore, while this study focused on ECG interpretation and referral patterns, it did not directly examine downstream diagnostic testing, imaging interpretation challenges, or clinical outcomes, including the potential for overdiagnosis or misdiagnosis in athletes. These factors may have influenced clinicians’ responses and warrant further prospective investigation. Finally, although qualitative data provided valuable insight into medico-legal concerns and system-level barriers, these perspectives remain context-specific and cannot be assumed to apply uniformly across Europe.

In addition, the distinction between sports cardiology specialists and nonspecialists was based on self-reported identification rather than predefined objective criteria such as formal training, accreditation, or procedural volume. This may have introduced misclassification bias and contributed to overlap between groups, which should be considered when interpreting comparisons between specialists and nonspecialists. The questionnaire was not formally validated, and no formal assessment of reliability or internal consistency was undertaken. As such, responses to subjective constructs such as confidence and familiarity should be interpreted with caution. This Survey reveals substantial European variability in ECG interpretation, referral pathways, training, and medico-legal governance in athlete care. It highlights the need for updated ECG criteria, harmonized sports cardiology training, and clearer medico-legal frameworks to improve the safety and consistency of cardiovascular screening in athletes.

## Conclusion

This international Survey provides a contemporary, real-world overview of how ECG interpretation and PPS are currently practised in athletes across Europe, among expert sports cardiologists and nonexpert medical professionals. It revealed substantial heterogeneity in access to services and training, as well as variability in ECG interpretation, linked to uncertainty. Furthermore, referral potentially involves suboptimal pathways and important medico-legal framework issues, as well as the challenges that arise from these. Supporting nonspecialists and addressing these discrepancies are crucial to improving clinical practice and ensuring athletes receive the most accurate and effective care. Future initiatives should focus on educational and training programmes to improve knowledge and confidence in interpreting athletes’ ECGs and mitigate the risks of misdiagnosis. This Survey timely provides essential clinician-informed justification for the evolution of the new criteria towards a more comprehensive framework that integrates ECG interpretation. This should include novel ECG biomarkers, with pragmatic clinical management recommendations, whilst supporting the role of advanced techniques including AI.

## Supplementary Material

oeag183_Supplementary_Data

## Data Availability

Data are available upon reasonable request. The anonymised data supporting the findings of this study may be made available by the corresponding author upon reasonable request, subject to applicable ethical, institutional and data protection requirements.
