# Supplementary material for: ECG interpretation in athletes by expert sports cardiologists and nonspecialist medical professionals across Europe: a survey from the European association of preventive cardiology
Source: Eur Heart J Open. 2026 Oct 29;6(6):oeag183. doi: 10.1093/ehjopen/oeag183 (PMC13739043; doi:10.1093/ehjopen/oeag183)
Supplement: Supplementary file 1 [file oeag183_supplementary_data.pdf]

Scope and Objectives of the Survey

The objective of this survey is to evaluate the level of incorporation of formal ECG criteria, by practitioners who are involved in athletic individuals' management. More specifically, this survey will evaluate:

- clinical practice and variations across European countries

- whether formal ECG criteria are actually used or clinicians are just based on their own experience

- whether various ECG parameters and guidance have been clear to the non-specialized clinician or there are misconceptions and 'misuse' of guidance

- whether sports discipline, athlete's age, gender, ethnicity and imaging parameters are taken into consideration when assessing athletic individuals

- whether there are gaps in evidence making specialized clinicians practice challenging

- whether an update for the specialized clinicians could assist and improve clinical practice

- the public awareness of European countries on this issue based on which there might emerge the need for raise awareness, creation of training programs, postgraduate programs, new sports cardiology centres accreditation referrals etc.

-And to explore potential barriers that may influence these variations across countries

EAPC Survey on Variability in interpretation of the Electrocardiogram in athletes based on International Criteria, across specialised and non-specialised clinicians

Disclaimer

Thank you for taking part in this survey. It should take no more than 3 minutes to complete. May we remind you of the codes of conduct related to market research among healthcare professionals:

- *We comply with the European General Data Protection Regulation (GDPR) 2016/679. Any personal data processed in connection with this survey will be treated confidentially and only used for the purpose of market research. Material and data collected from you will be analysed overall, used internally by the ESC and kept for a maximum of twenty-four months for analysis and quality control purposes. We take all reasonable care to prevent any unauthorised access to your personal data. We respect your privacy and your right to access, modify, or suppress your personal data. At any time, you can ask to know what personal data is being held. If you have any questions about data protection or require further information, please contact our data protection officer DPO@escardio.org*

- *This survey is anonymous. We will not disclose your identity to any third party You have the right to end your participation in this survey at any time*

Please confirm whether you have considered all the clauses above and accept to participate in this survey.

Yes / No

1. **Please select your age category (drop down)**

- 30-39
- 40-49
- 50-59
- 60-69
- 70 or older

2. **What is your gender? (drop down)**

- Female
- Male
- Other
- Do not want to say

3. **Which country do you practice in? (drop down)**

4. **Please select your place(s) of work**

- Hospital (Private)
- Hospital (Public)
- Private Practice
- Other (please specify)

5. **What is your speciality? (drop down)**
   a. Physician – General Cardiology [conditional logic : non-specialised]
   b. Physician – Sub-specialty in cardiology (heart failure, arrythmia, imaging, acute, prevention) [conditional logic : non-specialised]
   c. Sports Cardiologist [conditional logic : specialised]
   d. Sports Medicine specialist [conditional logic : specialised]
   e. Physician – Internal Medicine [conditional logic : non-specialised]
   f. Physician – General practice / Nurse [conditional logic : non-specialised]
   g. Other, please specify [conditional logic : non-specialised]

Page 2 - Non-specialised clinicians

1. **Is there a sports cardiology centre region?**

- Yes
- No

2. **Is there a sports specialist in your region?**

- Yes
- No

3. **Are you involved in the management of athletes (Athletes are individuals who compete in sports, requiring physical strength, speed, skill, and endurance.) ?**

- Yes
- No

4. **Conditional : if yes What types of Sports? (multiple choice)**

- Endurance
- Power
- Skill
- Mixed

5. **Do you frequently refer an athlete to the sports cardiologist? (Single choice)**

- I refer all athletes I see
- I refer most athletes
- I refer 50% of athletes
- I refer few athletes
- I never refer any athlete

6. **Are you familiar with the definition of an athletic individual and different types?**

Not at all familiar	Not really familiar	Neutral	Familiar	Very familiar

7. **Are you familiar with the different sports classification from the following societies?**

	Not at all familiar	Not really familiar	Neutral	familiar	Very familiar
European Association of Preventive Cardiology (EAPC)/European Society of Cardiology (ESC)					
Association for European Paediatric and Congenital Cardiology (AEPC)					
American Heart Association (AHA) /American College of Cardiology (ACC)					

8. **Are you familiar with the 2017 International ECG Criteria?**

- Yes
- No

9. **Conditional if yes = Do you use them in your everyday practice?**

- Yes
- No
- If no, could you please tell us why? Open text field (optional)

10. **How confident are you interpreting an athlete's ECG?**

Not at all confident	Not really confident	Neutral	Confident	Very confident

11. **Are you familiar with the findings below, according to the 2017 International ECG Criteria (multiple choice)?**

	Not at all familiar	Not very familiar	Neutral	Familiar	Very familiar
Normal findings					
Abnormal findings					
Borderline findings					

12. **Are you familiar with the different variations of T-wave inversions in 'anterior leads' requiring a potential referral to the specialist?**

	Not at all familiar	Not very familiar	Neutral	familiar	Very familiar
**juvenile pattern**					
**African Caribbean athlete**					
**Caucasian female**					
**ARVC**					

13. **Are you confident to refer for further investigations based on the 'ECG abnormality' or do you prefer to refer to the Sports specialist either way?**

- I am confident referring for further investigations
- I prefer referring to the sports specialist

14. Are you familiar with the concepts below (multiple choice)?

	Not at all familiar	Not very familiar	Neutral	familiar	Very familiar
Athletic adaptation					
Relevant ECG changes					
Overlap with certain cardiac pathologies					

15. Is there a cut-off level of bradycardia below which you would think it is likely beyond athletic adaptation and you would consider referring for further investigation?

- Yes
- No
- Unsure

16. Would you request further investigations for an athlete with voltage criterion for hypertrophy and left axis deviation?

- Yes
- No
- Unsure

17. Do you think the legal framework is efficient for the non-specialised clinician to make decisions related to the exclusion of athletic participation in your country?

- Yes
- No
- Unsure

18. Please describe in your own words :

A. the legal framework in your country: (open text field)

____

B. the challenges you are facing in your everyday practice (exercise prescription, communication with the club, Associations, Health Ministry, liability for exclusion an athlete or allowing to play in case of cardiac complication etc.) open text field

____

19. **Would you use a simple international guide for the non-specialist, to facilitate management and potential referral to the sports specialists, if it were available?**

Yes
No
If no, could you please tell us why? (open text field)

Page 3 - Specialised clinicians

1. **Is there a Sports Cardiology centre in your region?**

- Yes
- No

2. **In your daily practice, what is your approach to ECG interpretation?**

- I use the 'normal findings', 'abnormal findings' and 'borderline findings' classification, according to the 2017 International ECG Criteria
- I prefer a more flexible approach to ECG interpretation

3. **Do you think the following ECG changes are adequately described in the 2017 International ECG Criteria document?**

	Yes	No	Unsure
Anterior T-wave inversion' variations and their management			
LBBB and RBBB-like morphology and their management			
Ventricular ectopic beats morphology and management			

4. **Which cut-off level of ventricular extrasystoles per 24h would you use to consider further investigations in an athletic individual?**
   Add open field

5. **Do you think the ECG indices below and potentially related impact are adequately described in the 2017 International ECG Criteria document?**

	Yes	No	Unsure
QRS fragmentation			
small amplitude of QRS (for instance in inferior leads)			
non-specific intraventricular conduction delay			

6. **In your opinion, Is there adequate evidence to provide guidance on master athletes' ECG, which perhaps is not well represented in the current document? Yes/No/unsure**
   -Please explain

7. **In case of an 'abnormal ECG' Do you use the recommended further evaluation according to the 2017 International ECG Criteria, or do you prefer using a flexible approach in requesting further investigations based also on your experience?**

   a. I use the recommended further evaluation according to the 2017 international ECGCriteria.
   b. I prefer a flexible approach for further investigations based on my experience.

8. **In your opinion, is there is a significant degree of interobserver variability amongst Sports specialists?**

Strongly disagree	Disagree	Neutral	Agree	Strongly agree

9. **Are athletes' ECG interpretation and management mainly led by Sports specialists in your country? Yes/No/unsure**

10. **Conditional if yes : Do you think Sports specialists have adequate training and experience in your country? Yes/No/unsure**

11. **Do you think the legal framework in your country provides adequate guidance related to the acceptable medical risks of exercise participation of athletes with cardiovascular pathology?**

- Yes
- No
- Unsure

12. **Please describe in your own words :**
    a. **the Legal Framework in your country open text field**
    b. **challenges you are facing in your everyday practice (exercise prescription, communication with the club, Associations, Health Ministry, liability for exclusion an athlete or allowing to play in case of cardiac complication etc.). open text field**

13. **Based on the answers already provided, do you think there is a need for revision of the 2017 International ECG Criteria? (mandatory)**

- Yes
- No.
- Unsure
- Conditional logic : If yes or no could you please tell us why

14. **Do you think artificial intelligence could be of use in ECG interpretation in clinical practice? (mandatory)**

- Yes
- No
- Unsure

15. **In case of a future document, do you think there is a need to also include recent data on the use of artificial intelligence in ECG interpretation? (optional)**

- Yes
- No
- Unsure
